# Supplementary material for: Identification of photoperiod-induced specific miRNAs in the adrenal glands of Sunite sheep (Ovis aries)
Source: Front Vet Sci. 2022 Jul 22;9:888207. doi: 10.3389/fvets.2022.888207 (PMC9354845; doi:10.3389/fvets.2022.888207)
Supplement: Supplementary file 1 [file Data_Sheet_1.ZIP › Supplementary file/miRDeep2 file.zip!/miRDeep2 file/Novel_Structure/SP42A2/Novel_278.pdf]

Provisional ID	:	NC_019458.2_1903
Score total	:	2.5
Score for star read(s)	:	-1.3
Score for read counts	:	0
Score for mfe	:	2.2
Score for randfold	:	1.6
Score for cons. seed	:	
Total read count	:	246
Mature read count	:	246
Loop read count	:	0
Star read count	:	0

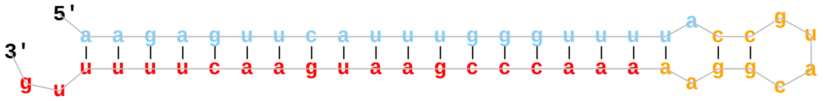

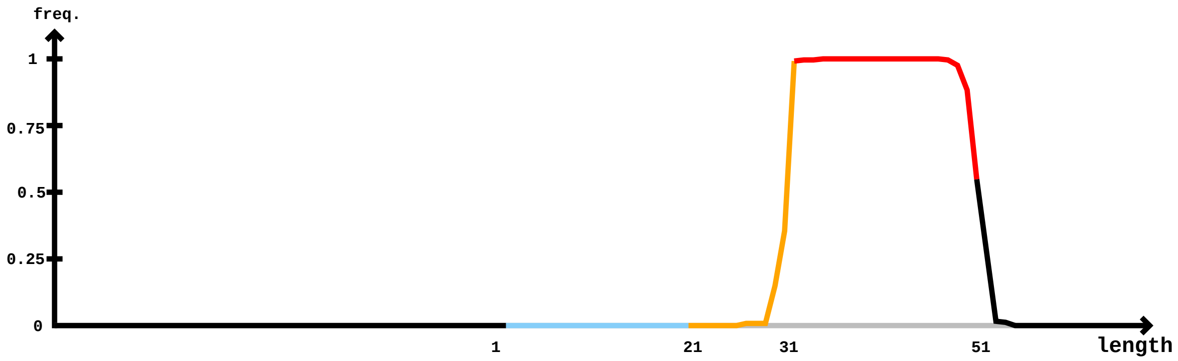

Star Mature

```
5'- uuaguccuugcagccacuuuaacaacagguccuuuggguuggccaaagaguucauuuggguuuuaccguacggaaaaacccgaaugaacuuuuuggcuaaucugauaccguc   -3'    exp
    ...........................(((...(((((((((((((((((((((((((((((((.((....)).))))))))))))))))))))))))))))))).)))...    reads    mm    sample
    ......................................................................cggaaaaacccgaaugaacuuu....................    1    0    seq
    ......................................................................cAgaaaaacccgaaugaacuuu....................    1    1    seq
    .........................................................................aaaaacccAaaugaacuu.....................    1    1    seq
    .........................................................................aaaaaccAgaaugaacuuuu...................    13    1    seq
    .........................................................................aaaaacccAaaugaacuuuu...................    1    1    seq
    .........................................................................aaaaacccgaaugaacuuuuG..................    14    1    seq
    .........................................................................aaaaaccAgaaugaacuuuuu..................    2    1    seq
    .........................................................................aaaaacccgaaugaacuuuuuA.................    1    1    seq
    .........................................................................aaaaacccgaaugaacuuuuug.................    1    0    seq
    .........................................................................aaaaaccAgaaugaacuuuuug.................    2    1    seq
    ..........................................................................aaaaccAgaaugaacuuu....................    2    1    seq
    ..........................................................................aaaacccgaaugaacuuu....................    1    0    seq
    ..........................................................................aaaacccgaaugaacuuuuu..................    3    0    seq
    ..........................................................................aaaaccAgaaugaacuuuuu..................    9    1    seq
    ..........................................................................aaaacccAaaugaacuuuuu..................    9    1    seq
    ..........................................................................aaaacccgaaugaacuuuuug.................    6    0    seq
    ..........................................................................aaaaccAgaaugaacuuuuug.................    2    1    seq
    ..........................................................................aaaacccgaaugaacuuuuuA.................    3    1    seq
    ..........................................................................aaaacccAaaugaacuuuuug.................    16    1    seq
    ...........................................................................aaacccgaaugaacuuuu...................    9    0    seq
    ...........................................................................aaacccgaaugaacuuuuu..................    45    0    seq
    ...........................................................................aaacccgaaugaacAuuuu..................    1    1    seq
    ...........................................................................aaacccgaaugaacuuuuuU.................    2    1    seq
    ...........................................................................aaacccgaaugaacuuuuug.................    85    0    seq
    ...........................................................................aaacccgaaugaacuuuuuC.................    2    1    seq
    ...........................................................................aaacccgaaugaacuuuuuA.................    11    1    seq
    ...........................................................................aaacccAaaugaacuuuuuggc...............    1    1    seq
    ...........................................................................aaacccgaaugaacuuuuuggcA..............    2    1    seq
    ............................................................................aacccgaaugaacuuuuuU.................    1    1    seq
    ..............................................................................cccgaaugaacuuuuuggcA..............    1    1    seq
```
